# Surface structure on diamond foils generated by spatially nonuniform laser irradiation

**DOI:** 10.1038/s41598-020-66036-3

**Published:** 2020-06-02

**Authors:** Hiroki Kato, Hideo Nagatomo, Mitsuo Nakai, Tatsuhiro Sakaiya, Hidenori Terasaki, Tadashi Kondo, Yoichiro Hironaka, Katsuya Shimizu, Keisuke Shigemori

**Affiliations:** 1 Institute of Laser Engineering, Osaka University, 2-6 Yamada-Oka, Suita, Osaka 565-0871 Japan; 2 Department of Earth and Space Science, Graduate School of Science, Osaka University, 1-1 Machikaneyama-Cho, Toyonaka, Osaka 560-0043 Japan; 3 Center for Science and Technology under Extreme Conditions, Graduate School of Engineering Science, Osaka University, 1-3 Machikaneyama-Cho, Toyonaka, Osaka 560-8531 Japan

**Keywords:** Condensed-matter physics, Plasma physics, Surfaces, interfaces and thin films

## Abstract

Here we report on the effects of material strength factors on the generation of surface structure due to nonuniform laser irradiation. The influence of material strength on the generation of perturbation on a diamond surface subjected to nonuniform laser irradiation was experimentally investigated. Our previous investigations suggested that stiffer and denser materials reduce surface perturbation due to spatially nonuniform laser irradiation, which was reproduced well by calculations with multi-dimensional hydrodynamic simulation code. In this work, we found that local fractures due to yield strength failure are generated by high degrees of irradiation non-uniformity. A characteristic crack-like surface structure was observed, which was not reproduced by the 2D simulation code calculations at all. The 2D simulations showed that the pressure at the diamond surface locally exceeds the Hugoniot elastic limit due to nonuniform irradiation, implying the potential for development of surface perturbations. We also measured the areal-density distribution of perturbations for single-crystal diamond and diamond with a thin high atomic number (high-Z) coating on its surface. The experimental results imply that the combination of a stiff material and thin high-Z coating can suppress the solid-strength effects caused by large irradiation non-uniformity. The knowledge given here is applicable to inertial confinement fusion target design, laser material processing, and universal problems involving solids and high-energy-density plasmas.

## Introduction

In direct-drive inertial confinement fusion (ICF)^1,2^, a fuel capsule is irradiated directly with laser light to achieve high-density compression. The capsule consists of a cryogenic layer of deuterium and tritium (DT) frozen onto the inner surface of a spherical shell of ablator material. Surface perturbation due to nonuniform irradiation occurs on the surface of the ablator material due to irradiation nonuniformity^3,4^. This spatial perturbation is amplified by Rayleigh-Taylor instability during the shell acceleration phase^5,6^, potentially disrupting the compressed shell and causing fuel mixing^7^. In the direct drive scheme, surface perturbation due to nonuniform irradiation is one of the most important issues, because imprinting a perturbation on the capsule surface degrades the symmetry of the compression. The level of surface perturbation due to nonuniform irradiation depends on the ignition condition parameters (neutron yield and target areal density) in ICF experiments^8^. Many previous investigations have striven to mitigate surface perturbation due to nonuniform irradiation by smoothing the effective laser irradiation nonuniformity or using low-density foam ablators^9–12^, high-Z coatings^13–15^ or high-Z dopants^16^. In our previous work, it was found that the use of stiffer and denser materials reduces the surface perturbation due to nonuniform irradiation^17^. Among the stiff materials, diamond is a top candidate as an ablator material for direct-drive ICF targets^17^. The advantages of stiff materials in ICF target design can easily combined with another suppression scheme, e.g., smoothing the effective laser irradiation nonuniformity or adding high-Z material coatings. Also, in x-ray indirect drive implosions, high-density carbon (HDC) is a leading candidate as an ablator material, because of the high implosion velocity and high stagnant pressure achievable due to its high density and optimal X-ray opacity^18^. Indirect-drive implosions with a HDC capsule are being conducted at the National Ignition Facility (NIF)^18–23^.

In the case of diamond, the shock physics is more complicated compared with conventional ICF capsule materials (e.g., plastic), due to its tightly bound crystalline structure in combination with an ultrahigh melting temperature, and the existence of the phase transition to BC8^24,25^ at ultrahigh pressure. The existence of a solid or partially melted diamond ablator during the ICF implosion phase can provide microstructures that seed hydrodynamic instabilities^18,26,27^. Using a strong shock that completely melts the diamond or keeping within the coexistence regime is necessary in order to suppress distortions of the shock front due to anisotropy in the sound velocity in crystals^18^. The anisotropy of diamond is indeed a concern for the ICF application, but recent experiments at NIF have demonstrated that this can be mitigated by using an optimized laser pulse profile^21^. Although the diamond with its high stiffness is becoming a candidate ablator material, brittle materials such as diamond can easily cleave due to dynamic stress along certain crystallographic planes^28,29^. In the case of direct-drive inertial confinement fusion, in particular, nonuniform laser irradiation would lead to local fracture on the brittle material surface. However, there has been no previous work done on material strength issues due to nonuniform irradiation so far.

In this paper, we report on the effects of material strength against dynamic stress on the surface perturbation due to spatially nonuniform laser irradiation. An understanding of the effects of solid strength under laser irradiation is also important in the basic physical process of laser material processing and the early phases of inertial confinement fusion. We carried out measurements of the areal density of perturbations due to non-uniform laser irradiation with the face-on x-ray backlighting method, which is a standard technique in hydrodynamic instability experiments^4,6^. In this study, we present perturbation areal-density data and their analysis, as well as calculations of perturbation areal density and amplitude with the two-dimensional hydrodynamic simulation code PINOCO-2D^30^. We analyzed the effects of irradiation nonuniformity and high-Z coating on the areal density of perturbations in diamond foils. All the results from the experiments and simulations suggest that material strength factors affect the surface perturbation due to nonuniform laser irradiation. In the Methods section, details of the experimental method and specifications for measurements of the areal density of perturbations are described.

## Results

### Measurements of perturbation areal-density growth with face-on backlighting technique

**Figure 1:**
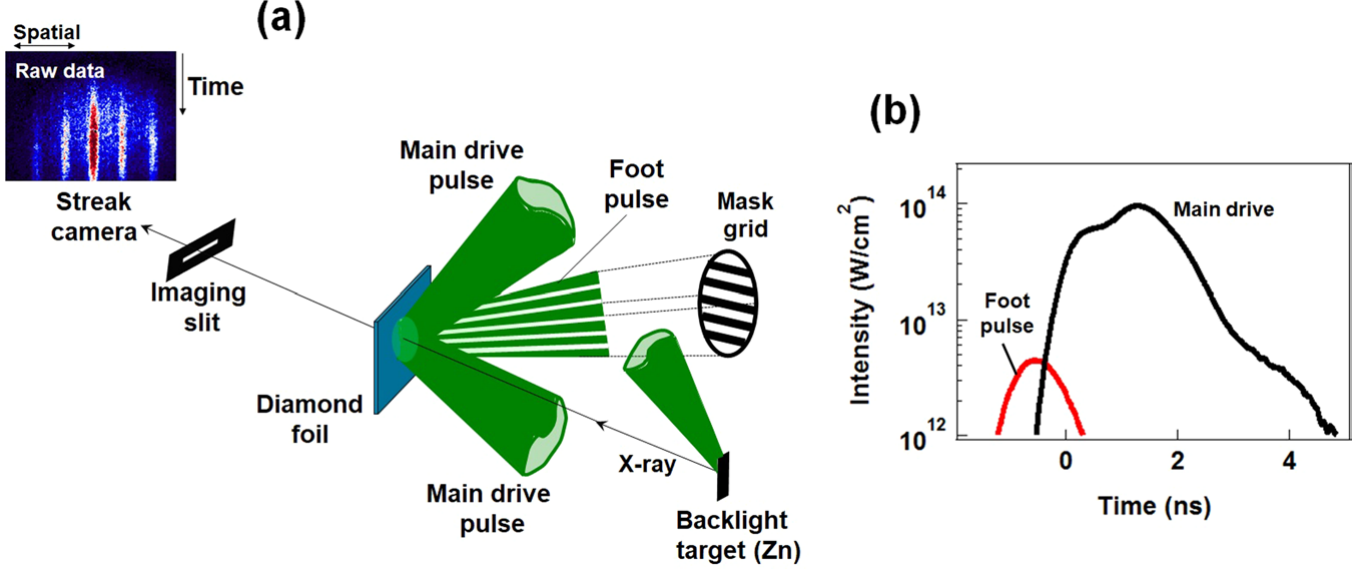
(**a**) Experimental setup for face-on x-ray backlighting measurement of areal-density perturbations seeded by nonuniform laser irradiation. (**b**) Typical laser pulse shape.

**Figure 2:**
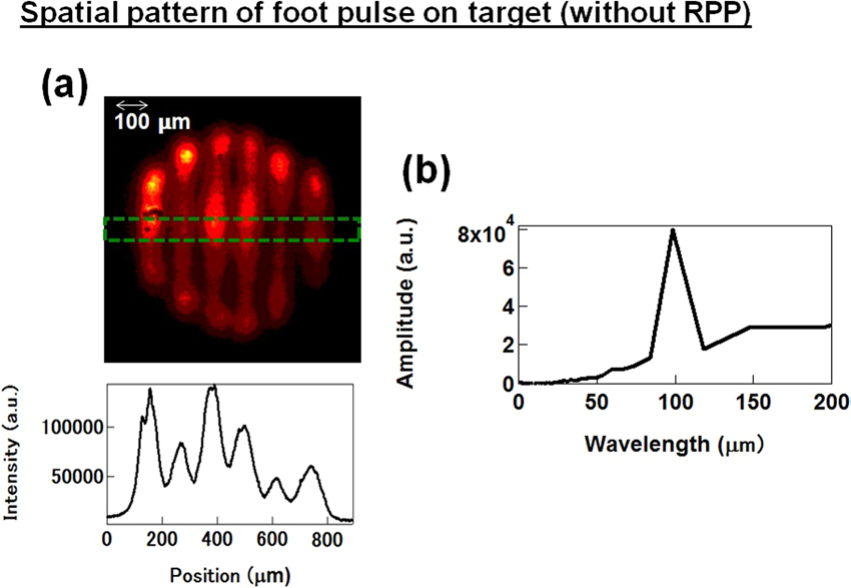
(**a**) Spatial pattern of foot pulse at target surface. (**b**) Modulation wavelength spectrum of irradiation non-uniformity.

The experiments were conducted using the GEKKO- XII Nd: glass laser facility at the Institute of Laser Engineering, Osaka University^31^. An overview of the experimental setup and typical stacked pulse shape are shown in Fig. 1. The diamond foils were irradiated with the second harmonic laser emission (wavelength: 0.527 μm). The stacked pulse with time delays between the beams consists of one beam for the foot pulse at an intensity of ~4.0 × 10^12^ W/cm^2^, and a subsequent pulse with two beams for the main drive pulse, at an intensity of ~10^14^ W/cm^2^ (see Fig. 1(b)). Figure 2(a) shows the spatial pattern of the foot pulse at the target surface. Foot pulses had irradiation nonuniformity (perturbation intensity/average intensity ~40%), in order to generate a surface perturbation due to nonuniform irradiation. The wavelength *λ* of the perturbation intensity variation was *λ* ~ 100 μm as shown in Fig. 2(b). Perturbations were observed on the target via amplification due to Rayleigh-Taylor instability (RTI) growth using the main drive beams, because the imprinted perturbations were typically too small for detection^15^.

**Figure 3:**
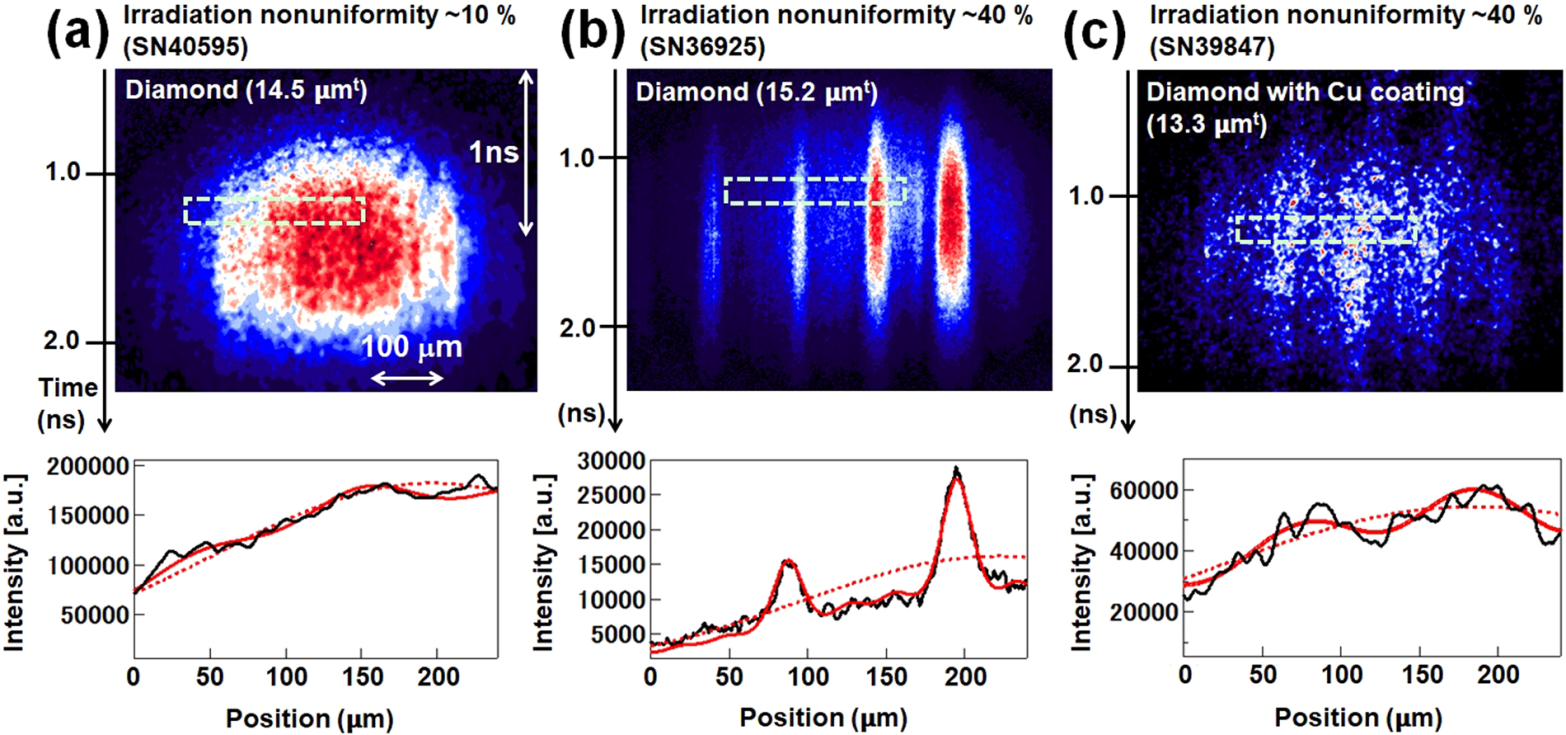
Raw streaked images of the backlit diamond (**a**) without Cu coating for irradiation nonuniformity ~10%, (**b**) without Cu coating for irradiation nonuniformity ~40%. (**c**) with Cu coating (0.1 μm^t^) for irradiation nonuniformity ~40%. All raw lineouts (black lines) are at about time 1.2 ns. Red lines are curve fits for each profile.

Examples of raw streaked backlit images of the diamond and diamond with thin Cu coating (0.1 μm^t^) foils for the foot pulse intensity ~4.0 × 10^12^ W/cm^2^ are shown in Fig. 3. The time origin (t = 0) was set as the time when the onset of the main drive pulse reached the half maximum, as shown in Fig. 1(b). Figure 3 also shows the lineouts for these targets. Time-integrated lineouts were obtained for the temporal resolution duration (~140 ps). The areal density of perturbations was obtained by fitting the convolutions of the resolution functions and sinusoidal perturbation functions to the raw lineouts, taking into account the x-ray absorption coefficient (at *hν* = 1.53 keV), which was calibrated with “cold” materials. This detail is described in the Methods section. From the lineouts for the diamond with irradiation non-uniformity ~10%, typical sinusoidal-like perturbations of the RTI growth can be seen in Fig. 3 (a). On the other hand, in Fig. 3 (b), the backlit image for the diamond with large irradiation non-uniformity of ~40% indicates a non-sinusoidal perturbation with “sharp” structure, which is different from the usual single-mode perturbation growth of the diamond observed for laser irradiation non-uniformity ~10%^17^. Please note that the structure on the diamond surface is referred to as “crack”-like because we observed sharp grooves from the shadowgraph measurements. When a high-Z coating was present on the diamond, no sharp structure was observed as in Fig. 3 (c); instead, the non-uniformity was close to the typical sinusoidal perturbations of the RTI growth.

**Figure 4:**
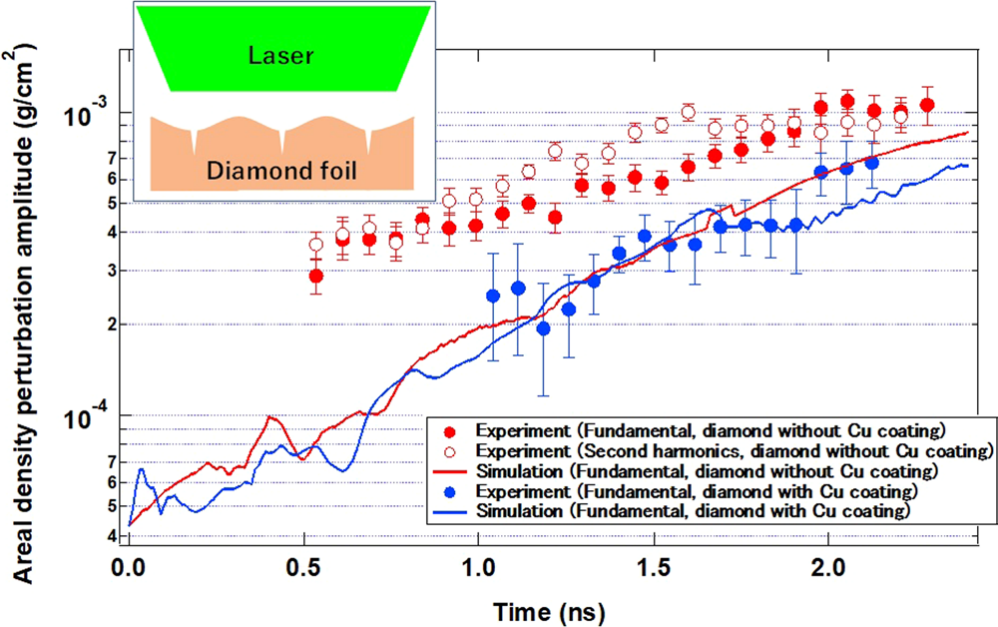
Areal-density perturbation growth for diamond targets from experiments on fundamental and second harmonic components (symbols) and from the PINOCO-2D simulations (solid curves) for each experimental configuration: (red) diamond on non-uniformity ~40%, (blue) diamond with Cu coating on non-uniformity ~40%. Also shown are schematics of crack-like surface structure for the diamond foil without Cu coating.

Figure 4 presents plots of the temporal evolution of the areal density of perturbations for the diamond with laser irradiation ~40%, with the fundamental (*λ*: 100 μm) and second harmonic (*λ*: 50 μm) perturbation. In our previous work^17^, for diamond with irradiation nonuniformity ~10%, it was found that only the fundamental perturbation was observed, which was well-reproduced by the two-dimensional radiation hydrodynamic simulation code PINOCO-2D^30^. For the diamond with large irradiation non-uniformity of ~40%, however, the second harmonic component rises to the same level as the fundamental component at very early times, prior to onset of the foil acceleration: ~0.75 ns. Both fundamental and second harmonic components individually grow with their own growth rates. For the diamond with large irradiation nonuniformity of ~40%, ordinal imprinting generation and amplification are not observed, unlike previous studies of single spatial mode planar foil experiments^4,6^. Also shown are the results of calculation with the PINOCO-2D code, which do not reproduce the experimental result at all. The second harmonic component from the PINOCO-2D simulation is negligibly small compared with the fundamental component.

The temporal evolution of the areal density of perturbations for diamond with a Cu coating with irradiation non-uniformity of ~40% is also plotted in Fig. 4. The fundamental perturbation grew with time, which is in good agreement with the PINOCO-2D simulation result. The growth with time of the second harmonic perturbation is much smaller than that of the fundamental component after the foil acceleration time of ~0.5 ns (not shown here). Hence, a sinusoidal-like perturbation arises, as seen in Fig. 3(c).

Please note that the second harmonic components without Cu coating are antiphase from those with Cu coating. The second harmonic generation on the Cu-coated diamond is very typical Rayleigh-Taylor instability in the linear to non-linear growth regime, which is called bubble-spike generation. On the other hand, the perturbation shape on the diamond is not the bubble-spike shape but rather the “crack”-like structure, as illustrated in Fig. 4. This fact clearly shows that some hidden physics exists in the generation of imprinting perturbations on the diamond surface.

## Discussion

**Figure 5:**
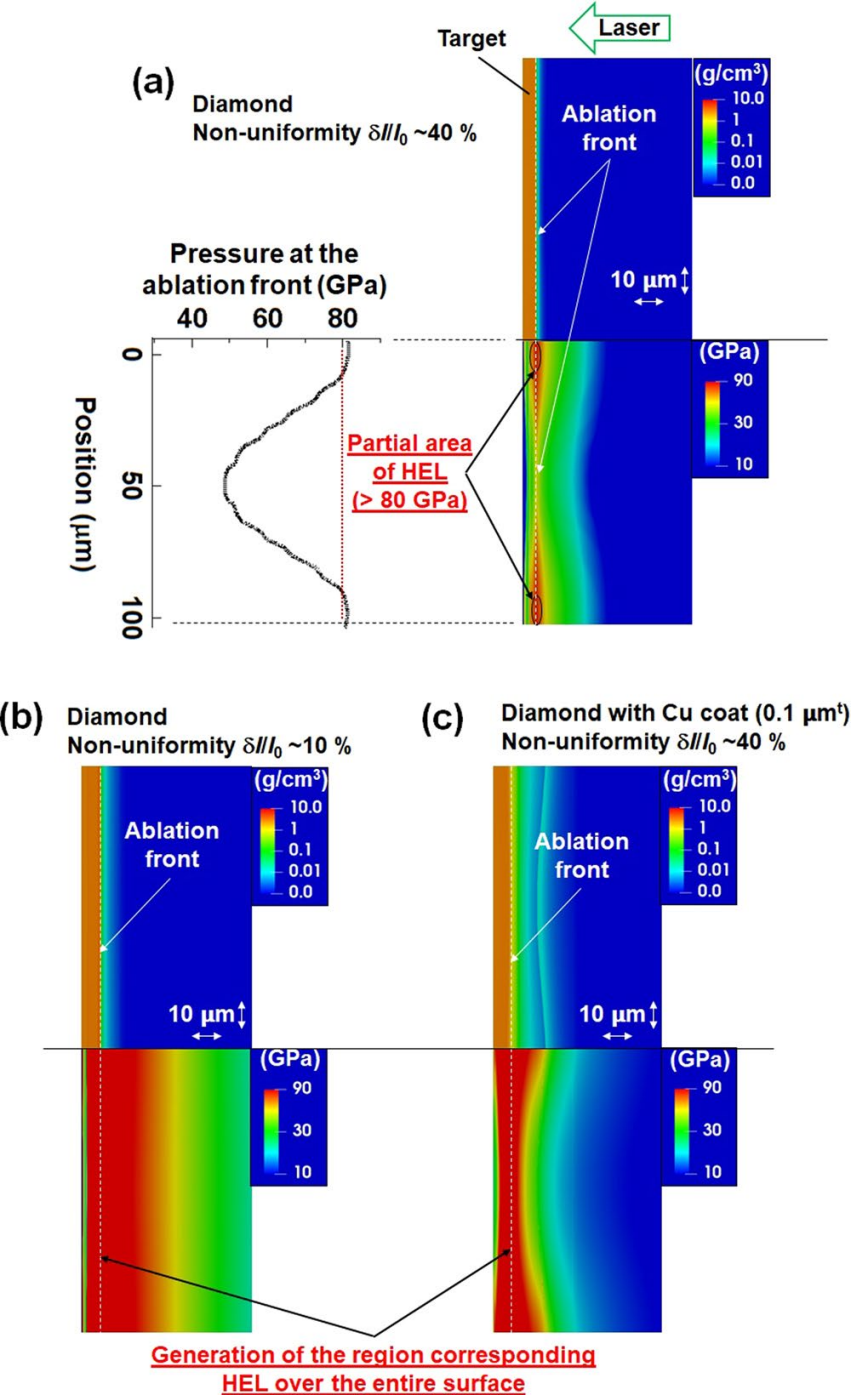
Simulated density and pressure contour plots for diamond foils at −0.95 ns: (**a**) Nonuniformity ~40%. (**b**) Nonuniformity ~10%. (**c**) Nonuniformity ~40% with Cu coating.

From the experimental results, the 2D hydrodynamic simulation calculations do not reproduce the experimental results when the irradiation non-uniformity is large, as shown above. In order to interpret these facts, we consider the effects of materials strength on the generation of surface perturbation due to nonuniform laser irradiation. From previous shock compression experiments, the Hugoniot elastic limits (HEL) of diamond are measured to be 80.1 (±12.4), 80.7 (±5.8) and 60.4 GPa (±3.3) for the <100>, <110>, and <111> orientations, respectively^32^. The elastic yield strength of diamond inferred from these measurements is 75 (±20) GPa^32^. In our experiments, pressure perturbations due to the foot pulse produce non-uniform stress, namely tensile stress and shear stress, in the diamond foils. In the regime beyond HEL pressures, slip and fractures, which are not taken into account in the hydrodynamic simulation code, may occur primarily on crystal planes^32,33^. As a result, non-sinusoidal perturbation would be generated at the diamond surface. Figure 5 shows the density and pressure distribution calculated by the PINOCO-2D code near the ablation front at early irradiation times. When the irradiation nonuniformity is large (40%), a compressed area exceeding the HEL or (elastic yield strength) of diamond locally appears as shown in Fig. 5(a). On the other hand, when irradiation nonuniformity is small (10%), most of the compressed area is over the HEL (Fig. 5(b)). The experimental data clearly indicate that the spatial perturbation is generated on the ablation surface (compression region), then the areal-density perturbation grows due to the Rayleigh-Taylor instability. Thus, the presence of crack-like structure in this experiment is not explained by cavitation nor spallation that occur when the pressure is released. At the pressure over the HEL, crystal is collapsed and transformed to plastic state which is movable like a fluid. On the other hand, crystal at elastic state is difficult to move. When the irradiation nonuniformity is large (40%), there appears two regions: elastic and plastic states on the diamond surface. Therefore, the mechanical local fracture due to elastic-plastic transition is the most probable interpretation for the generation of the crack-like structure.

We have also evaluated the effect of material strength using a thin high-Z coating that smooths the effects of irradiation non-uniformity or pressure perturbation. In previous multi-mode imprint experiments^13–15^, under initial low-intensity laser irradiation, the high-Z ablation layers were observed to expand and convert the initial nonuniform laser flux into uniform x-ray radiation, which uniformly ablates and accelerates the target. As the laser pulse shifts to higher intensities, the high-Z material burns away, and the target transitions to pure direct drive^13–15^. Figure 5(c) shows the pressure distribution for the non-uniform foot pulse irradiation (40%) for the diamond with a Cu coating. It is clear that pressure perturbation can be reduced by the high-Z coating compared to that without a Cu coating. That means local fracture at the surface is suppressed due to relaxation of the pressure perturbation. Hence the influence of material strength would be suppressed by the presence of a high-Z coating. As shown in Fig. 3, the crack-like structure, which is a non-sinusoidal perturbation, disappears for the samples coated by thin high-*Z* layers. This also suggests that the combination of diamond and high-Z coating are effective for suppression of the material strength effects due to large irradiation non-uniformity.

In conclusion, we have investigated the solid-strength effects on surface perturbation due to nonuniform laser irradiation when using diamond as an ablator material for ICF targets. When the irradiation non-uniformity is large, a compressed area exceeding the HEL of diamond develops, showing a crack-like surface structure. The thin high-Z surface coating is effective in suppressing local fracture due to the large pressure perturbation. These findings are particularly closely related to the target physics at the early irradiation time regime in direct-drive ICF. In particular, the performance of laser implosion is sensitive to fracture of the brittle ablator due to nonuniform irradiation. The knowledge from this study is crucial to the understanding not only of ICF target physics but also laser-material interactions and laser material processing. Further investigations on more precise measurements and modeling would reveal the detailed physics of the structure on laser irradiated surface.

## Methods

### Target samples and laser conditions

The targets comprised single-crystal diamond foils (Type-Ib, density: 3.51 g/cm^3^) with a thickness of 13–16 μm^17^. The surface orientation of the single-crystal diamond was the (100) plane. The target foils were coated with Al of 0.05 μm thickness as a shield for shine-through inside of the foils in the very early irradiation time regime. Some of the diamond foils were surface-coated with Cu of 0.1 μm thickness in order to compare the smoothing effects on irradiation non-uniformity, which is described above in the Results and Discussion sections.

The diamond foils were irradiated with the second harmonic laser emission (Wavelength = 0.527 μm) at an incident angle of 37.4°. The laser pulse consisted of one beam for the foot pulse and two beams for the main drive pulse, with time delays between the beams (Fig. 1(b)). The pulse shape was Gaussian with a 1.3 ns duration at full width at half maximum (FWHM). The laser pulse was focused on to the diamond foil to a spot with a size of ~600 μm (FWHM). The average intensity, *I*_0_, of the foot pulse was ~4 × 10^12^ W/cm^2^. The peak intensity of the main drive was ~10^14^ W/cm^2^. Intensity modulation *δI* of the foot pulse was introduced using a grid mask placed in front of the focusing lens, whereas the main drive was kept uniform. The spatial pattern and intensity distribution of the foot pulse on the target are shown in Fig. 2(a). The modulation wavelength on the target surface was 100 μm, with intensity non-uniformity *δI*/*I*_0_ ~ 10% or ~40%. The higher spatial harmonic components (wavelength: 20–50 μm) in the imprint pulse were less than 10% of the fundamental wavelength (as shown in Fig. 2(b)).

### Measurements of areal-density perturbation with face-on x-ray backlighting technique

Perturbations were observed on the target via amplification due to the RTI growth using the main drive beams because the imprinted perturbations were typically too small for detection^17^. The areal-density perturbation growth was measured using a face-on x-ray backlighting technique. A backlight target (Zn) was irradiated to generate ~1.53 keV quasi-monochromatic x-rays coupled with a 6 μm-thick aluminum filter. Temporal evolution of the transmitted x-rays from the Zn backlight target through a diamond foil was imaged through a slit (10 × 50 μm^2^) onto the CuI photocathode of an x-ray streak camera. The total magnification was ~25.9×, and the temporal resolution of the x-ray streak camera was ~140 ps.

### Data analysis

The spatial resolution was measured using a backlit grid image that took into account the analysis of the areal-density perturbation. The Au mesh (63.5 μm/period) was used to obtain the backlit grid image. The resolution function *R*(x) of the entire diagnostics system is given by the sum of two Gaussian functions as

1 $$R(x)=\left(\frac{1}{\sqrt{2\pi }}\right)\left[\frac{1}{({\sigma }_{1}+\alpha {\sigma }_{2})}{e}^{\left(-,\frac{{x}^{2}}{2{{\sigma }_{1}}^{2}}\right)}+\frac{\alpha }{({\sigma }_{1}+\alpha {\sigma }_{2})}{e}^{\left(-,\frac{{x}^{2}}{2{{\sigma }_{2}}^{2}}\right)}\right],$$

where *α* = 0.242, *σ*_1_ = 4.881 μm, and *σ*_2_ = 11.303 μm. The areal-density perturbations *δ*(*ρl*) were obtained by fitting the convolution of the resolution and a sinusoidal perturbation function to the raw lineouts, taking into account the x-ray absorption coefficient (*μ* = 660.9 cm^2^/g) for diamond. The raw lineouts function *I*(x) is expressed as

2 $$I(x)=\int R(x-u){I}_{0}(u){e}^{-\mu \{\delta {(\rho l)}_{1}\cos (ku)+\delta {(\rho l)}_{2}\cos (2ku)\}}du,$$

where *I*_0_ is the spatial intensity distribution of the backlight X-ray source, and *δ*(*ρl*)_1_ and *δ*(*ρl*)_2_ are perturbation amplitudes of the fundamental component and the second harmonic component, respectively. *k* (2*π*/perturbation wavelength) is the wave number of the raw lineouts. Fitting by convolution, considering the fundamental and second harmonic components, was in good agreement with the line profile. The trajectories of the irradiated foils were also determined using side-on x-ray backlighting in order to evaluate their basic hydrodynamics.

Simulations were carried out using the two-dimensional (2D) radiation hydrodynamic code PINOCO-2D for comparison with the three experimental configurations. PINOCO-2D gives the arbitrary Lagrangian Eulerian (ALE) hydrodynamic for the radiation. This code includes hydrodynamic, flux-limited Spitzer-Härm thermal conduction^34,35^, nonlocal thermal equilibrium multigroup radiation transport, quotidian equation of state^36^, and ray-trace laser-energy deposition. For the EOS, we incorporated a multiphase EOS^37^, and a table of melting curves^38^ for diamond with the quotidian equation of state model. Please note that the combined EOS model does not include local fracture nor crack progress.

The areal-density perturbations δ(ρl) of the 2D simulation are obtained from $\delta \rho l=\delta (\int \rho dx)={\int }_{{x}_{r}({y}_{p})}^{{x}_{a}({y}_{p})}\rho (x,{y}_{p})dx-$ ${\int }_{{x}_{r}({y}_{u})}^{{x}_{a}({y}_{u})}\rho (x,{y}_{u})dx.$Here, the x axis is perpendicular to the target surface, *x*_a_ is the position of the ablation front, *x*_r_ is the position of the rear surface, *ρ*(*x,y*) is density distribution in the target, and *y*_p_ and *y*_u_ are the perturbed and unperturbed y coordinates of the transverse direction, respectively. The ablation front x_a_ and rear front *x*_r_ are defined to be at 1/e of the peak density. In our calculation of perturbation areal density, the density distribution *ρ*(*x,y*) is considered as mentioned above.

## References

[1] Craxton RS (2015). Direct-drive inertial confinement fusion: A review. Phys. Plasmas.

[2] Atzeni, S. & Meyer-Ter-Vehn, J. The Physics of Inertial Fusion (Clarendon Press, Oxford, 2004).

[3] Ishizaki R, Nishihara K (1997). Propagation of a rippled shock wave driven by nonuniform laser ablation. Phys. Rev. Lett..

[4] Nakai M (2002). Single spatial mode experiments on initial laser imprint on direct-driven planar targets. Phys. Plasmas.

[5] Bodner SE (1974). Rayleigh-Taylor instability and laser-pellet fusion. Phys. Rev. Lett..

[6] Azechi H (1997). Direct-drive hydrodynamic instability experiments on the GEKKO XII laser. Phys. Plasmas.

[7] Bodner SE (1998). Direct-drive laser fusion: Status and prospects. Phys. Plasmas.

[8] Nora R (2014). Theory of hydro-equivalent ignition for inertial fusion and its applications to OMEGA and the National Ignition Facility. Phys. Plasmas.

[9] Desselberger M, Jones MW, Edwards J, Dunne M, Willi O (1995). Use of x-ray preheated foam layers to reduce beam structure imprint in laser-driven targets. Phys. Rev. Lett..

[10] Watt RG (1998). Laser imprint reduction using a low-density foam buffer as a thermal smoothing layer at 351-nm wavelength. Phys. Rev. Lett..

[11] Metzler N, Velikovich AL, Schmitt AJ, Gardner JH (2002). Laser imprint reduction with a short shaping laser pulse incident upon a foam-plastic target. Phys. Plasmas.

[12] Depierreux S (2009). Laser Smoothing and Imprint Reduction with a Foam Layer in the Multikilojoule Regime. Phys. Rev. Lett..

[13] Obenschain SP (2002). Effects of thin high-Z layers on the hydrodynamics of laser-accelerated plastic targets. Phys. Plasmas.

[14] Karasik M, Weaver JL, Aglitskiy Y, Oh J, Obenschain SP (2015). Suppression of Laser Nonuniformity Imprinting Using a Thin High-Z Coating. Phys. Rev. Lett..

[15] Mostovych A (2008). Enhanced Direct-Drive Implosions with Thin High-Z Ablation Layers. Phys. Rev. Lett..

[16] Hu SX (2012). Mitigating laser imprint in direct-drive inertial confinement fusion implosions with high-Z dopants. Phys. Rev. Lett..

[17] Kato H., Shigemori K., Nagatomo H., Nakai M., Sakaiya T., Ueda T., Terasaki H., Hironaka Y., Shimizu K., Azechi H. (2018). Effect of equation of state on laser imprinting by comparing diamond and polystyrene foils. Physics of Plasmas.

[18] MacKinnon AJ (2014). High-density carbon ablator experiments on the National Ignition Facility. Phys. Plasmas.

[19] Ross JS (2015). High-density carbon capsule experiments on the national ignition facility. Phys. Rev. E.

[20] Meezan NB (2015). Cryogenic tritium-hydrogen-deuterium and deuterium-tritium layer implosions with high density carbon ablators in near-vacuum hohlraums. Phys. Plasmas.

[21] Berzak Hopkins LF (2015). First High-Convergence Cryogenic Implosion in a Near-Vacuum Hohlraum. Phys. Rev. Lett..

[22] Olson RE (2016). First Liquid Layer Inertial Confinement Fusion Implosions at the National Ignition Facility. Phys. Rev. Lett..

[23] Le Pape S (2018). Fusion Energy Output Greater than the Kinetic Energy of an Imploding Shell at the National Ignition Facility. Phys. Rev. Lett..

[24] Biener J (2009). Diamond spheres for inertial confinement fusion. Nucl. Fusion.

[25] Johnston RL, Hoffmann R (1989). Superdense carbon, C8: supercubane or analog of γ-silicon?. J. Am. Chem. Soc..

[26] Celliers PM (2010). A high-resolution two-dimensional imaging velocimeter. Rev. Sci. Instrum..

[27] Landen OL (2008). Experimental studies of ICF indirect-drive Be and high density C candidate ablators. J. Phys. Conf. Ser..

[28] Field, J. E. The Properties of Natural and Synthetic Diamond (Academic, London, 1997).

[29] Telling RH, Pickard CJ, Payne MC, Field JE (2000). Theoretical Strength and Cleavage of Diamond. Phys. Rev. Lett..

[30] Nagatomo H (2007). Optimization of cone target geometry for fast ignition. Phys. Plasmas.

[31] Yamanaka C (1981). Nd-doped phosphate glass laser systems for laser-fusion research. IEEE J. Quantum Electron..

[32] McWilliams RS (2010). Strength effects in diamond under shock compression from 0.1 to 1 TPa. Phys. Rev. B.

[33] Eggert, J. H. *et al*. Anisotropic Shock Propagation in Single Crystals in Proceedings of Joint 20th AIRAPT-43th EHPRG International Conference on High Pres- sure Science and Technology, Karlsruhe, 2005.

[34] Boehly TR (2001). Optical and plasma smoothing of laser imprinting in targets driven by lasers with SSD bandwidths up to 1 THz. Phys. Plasmas.

[35] Spitzer L, Härm R (1953). Transport Phenomena in a Completely Ionized Gas. Phys. Rev..

[36] More RM, Warren KH, Young DA, Zimmerman GB (1988). A new quotidian equation of state (QEOS) for hot dense matter. Phys. Fluids.

[37] Benedict LX (2014). Multiphase equation of state for carbon addressing high pressures and temperatures. Phys. Rev. B.

[38] Wang X, Scandolo S, Car R (2005). Carbon Phase Diagram from Ab Initio Molecular Dynamics. Phys. Rev. Lett..

